# Development and testing of a smart empowerment education system for gout patients in suburban areas

**DOI:** 10.3389/fpubh.2026.1753575

**Published:** 2026-05-01

**Authors:** Tao Jiang, Liyuan Ge, Xinguo Wang, Lifeng Zhou

**Affiliations:** 1 School of Humanities and Management, Guilin Medical University, Guilin, China; 2 School of Public Health, Guilin Medical University, Guilin, China; 3 College of Public Health, Shanghai University of Medicine and Health Sciences, Shanghai, China; 4 Huinan Community Health Service Center, Shanghai, China

**Keywords:** empowering education, gout, refined management, self-management, suburban areas

## Abstract

**Objectives:**

This study aimed to develop and evaluate an Intelligent Empowerment Education-based System (IEES), which integrates empowerment education, refined management protocols, and smart technologies, for application among gout patients in resource-limited suburban settings. The primary goal was to address key challenges including suboptimal self-management capacity, poor treatment adherence, and unfavorable long-term clinical outcomes.

**Methods:**

A smart gout management system was developed using a SpringBoot+Vue framework, incorporating deep learning algorithms for personalized recommendations. A total of 90 gout patients admitted to a community health service center from January 2023 to December 2023 were randomly divided into a control group (*n* = 45) and an observation group (*n* = 45) according to a random number table. The control group received routine outpatient health education, while the observation group received an additional intervention based on empowerment education combined with refined management.

**Results:**

The system was successfully developed with key performance indicators including response time <200 ms and prediction accuracy of 89.7%. The self-management ability score of the observation group was (145.6 ± 4.5), significantly higher than that of the control group (*p* < 0.05). The fasting serum uric acid (SUA) value of the observation, and the liver and kidney function were significantly lower than those of the control group (*p* < 0.05).

**Conclusion:**

The intelligent empowerment education disease management system significantly improved SUA control, self-management ability, and renal function in suburban gout patients. This “Internet+” chronic disease management model demonstrates promise for scalable application.

## 1 Introduction

Gout, a metabolic disorder characterized by hyperuricemia, poses a significant global public health challenge. Its prevalence has been steadily increasing worldwide, currently ranging from 0.03 to 15.30% (1). In China, the overall prevalence has reached 1.1% (2), contributing substantially to the healthcare burden. Effective long-term management of gout is crucial to prevent complications such as joint disability, renal stones, and impaired kidney function. However, achieving sustained serum uric acid (SUA) control remains difficult, with only about one-third of patients globally reaching target SUA levels (3), and a reported 38.20% achievement rate in China after 6 months of treatment (4). This highlights a significant gap in current care paradigms (5).

Self-management is a cornerstone of effective gout control, yet studies consistently show that patients’ self-management capabilities are often suboptimal (6, 7). The Empowerment Education model, pioneered by Funnell (8), shifts the dynamic of care by placing the patient at the center, fostering autonomy, and enhancing self-efficacy (9–11) While promising, traditional empowerment education can be resource-intensive, difficult to standardize, and challenging to sustain.

The evolution of patient education reflects a paradigm shift from didactic information transfer towards fostering self-efficacy and autonomy. While traditional models often struggle with long-term adherence, empowerment education, as conceptualized by Funnell et al., places the patient at the center of care, emphasizing collaborative goal-setting and problem-solving (8). Translating this theory into scalable practice remains challenging. Digital health technologies offer unprecedented tools for delivering personalized, interactive, and sustained education. However, their design frequently lacks grounding in robust behavioral theory, limiting their effectiveness (12). Thus, a critical research gap exists in operationalizing empowerment theory through a structured digital framework to create a replicable and effective patient education strategy for chronic disease management.

Concurrently, refined management principles emphasize precision, detail, and comprehensive process control (13), offering a framework for systematic intervention but often lacking personalized, dynamic adaptation (14).

The integration of modern information technologies such as the Internet of Things (IoT), Artificial Intelligence (AI), and Big Data presents a transformative opportunity for chronic disease management. “Internet+” healthcare models can facilitate remote monitoring and education. AI and Deep Learning algorithms can analyze complex patient data to generate personalized lifestyle and medication recommendations, moving beyond one-size-fits-all approaches. IoT devices enable real-time collection of physiological and behavioral data, providing a continuous stream of information for Big Data analytics. This allows for the identification of patterns, prediction of gout flares (e.g., using LSTM networks), and dynamic adjustment of intervention strategies, creating a truly Smart Healthcare solution.

Pilot studies on multidisciplinary gout programs have shown promise (6). However, these initiatives often fall short of fully leveraging an integrated, technology-driven empowerment approach. When examining the translation of established concepts into practice, three significant gaps become apparent, particularly for underserved populations such as those in suburban areas.

First, while digital health tools for gout exist, they frequently remain fragmented, focusing on isolated functions like medication tracking or passive education, rather than operationalizing a comprehensive theoretical model into a cohesive digital workflow necessary for sustained behavior change (15, 16). Second, a pronounced equity gap persists; most solutions are developed in well-resourced, urban settings, leaving their effectiveness for communities with limited access, lower digital literacy, and distinct lifestyles severely understudied, which exacerbates health disparities (17, 18). Third, the synergistic potential of IoT, AI, and behavioral theory is largely unrealized. IoT often stops at data collection, and AI predictions are rarely embedded within a proactive patient empowerment framework, missing the shift from data aggregation to theory-informed, adaptive personalization (19, 20).

However, beyond these technical and design gaps, a critical translational challenge persists. Many digital health interventions, even those demonstrating efficacy in controlled research settings, fail to generate lasting impact in routine practice due to a lack of grounding in implementation science frameworks and explicit plans for long-term sustainment (21, 22). There remains a paucity of evidence on how to systematically operationalize and sustain theory-driven, technology-facilitated interventions within the constraints of real-world community healthcare systems, particularly for underserved populations (23). This limits the development of generalizable and scalable management strategies from digital health innovations.

To address these interconnected gaps, we hypothesized that a system which codifies the patient-centered philosophy of empowerment education and the precision of refined management into a robust intelligent technology platform—integrating IoT, Deep Learning (AI), and Big Data analytics—would overcome the limitations of both traditional and existing digital methods (24–26). We posited that this Intelligent Empowerment Education System (IEES) would provide personalized, accessible, and sustained support specifically tailored for suburban gout patients, thereby leading to superior improvements in self-management behaviors and clinical outcomes (27–31).

## 2 Methods

### 2.1 Randomization and blinding

The random sequence was generated by an independent statistician using a computer-generated random number table. Allocation concealment was implemented using the sealed envelope method. Group assignment was carried out by a research assistant not involved in the intervention. Due to the nature of the intervention, blinding of participants and intervention providers was not possible. However, outcome assessors (laboratory technicians and data analysts) were blinded to group allocation.

### 2.2 Participants

**Figure 1:**
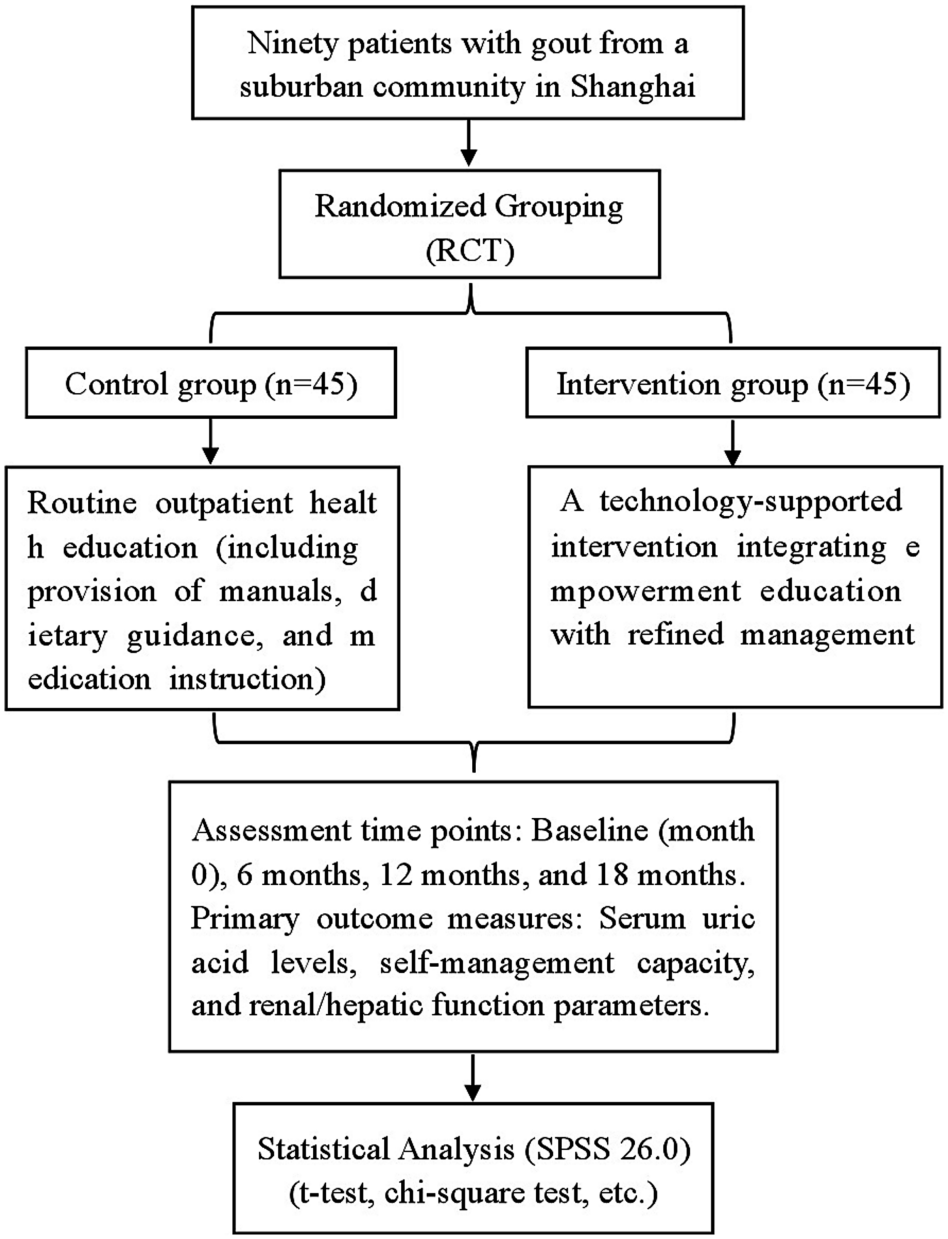
Study flow diagram.

This study used a convenience sampling method to select 90 gout patients from the outpatient department of a community health service center in the Shanghai suburbs between January 2023 and December 2023 (Figure 1). Inclusion criteria: ① Diagnosed with gout according to the 2015 ACR/EULAR gout classification criteria (27); ② Age 18–75 years; ③ Mentally clear and independently mobile; ④ Voluntarily participated and provided informed consent. Exclusion criteria: ① Secondary gout caused by drugs, malignant tumors, radiotherapy/chemotherapy, endocrine, or renal diseases; ② Severe diseases affecting vital organs such as the heart, liver, or kidneys; ③ Pregnant or lactating women; ④ Severe mental illness or cognitive dysfunction; ⑤ Poor compliance, unwilling to cooperate. Elimination criteria: ① Voluntary withdrawal; ② Inability to continue participation due to severe illness. Using a random number table, the 90 subjects were divided into a control group (*n* = 45) and an observation group (*n* = 45). There were no statistically significant differences in general characteristics between the two groups (*p* > 0.05), indicating comparability (Table 1). The study was approved by the Ethics Committee.

**Table 1:** General information of the control group and the observation group.

Group	Age (X ± s)	Gout Course (Years, X ± s)	Male (n/%)	Education ≥ High School (n/%)	Patients with complications (n/%)			
					Hypertension	Hyperlipidemia	Diabetes	Coronary heart disease
Control	46.9 ± 16.4	5.1 ± 5.3	38(84.4)	25(55.6)	14(31.1)	5(11.1)	9(20.0)	2(4.4)
Observation	47.5 ± 16.3	5.2 ± 4.8	39(86.7)	23(51.1)	16(35.6)	3(6.7)	8(17.8)	1(2.2)

### 2.3 Flow of participants through the study

**Figure 2:**
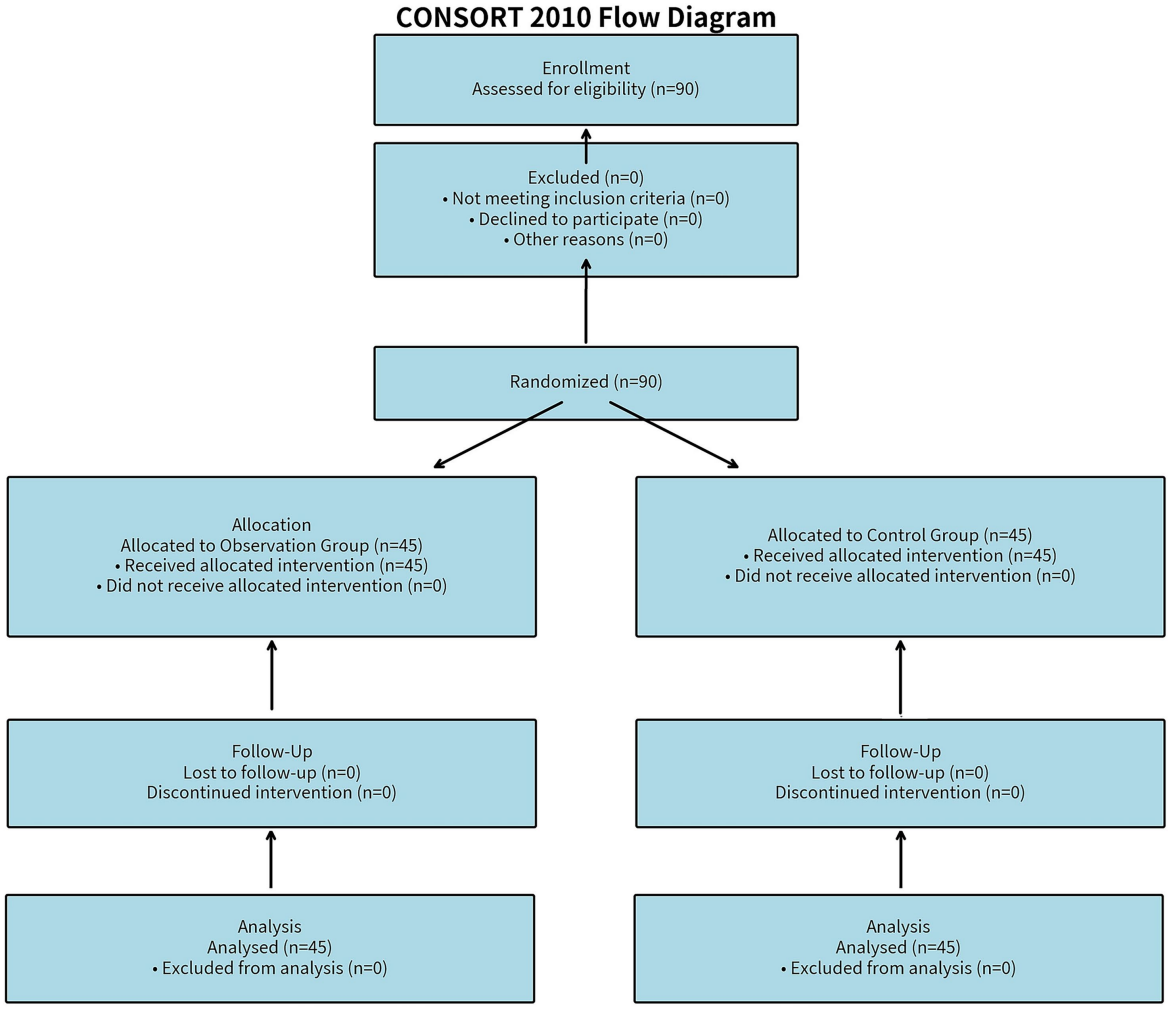
Flow of participants through the study.

The flow of participants through the study is presented in Figure 2 (CONSORT 2010 flow diagram). A total of 90 gout patients were assessed for eligibility and subsequently enrolled. No participants were excluded prior to randomization. All 90 participants were randomly allocated to either the observation group (*n* = 45) or the control group (*n* = 45). All participants in both groups received their allocated interventions. During the 6-month intervention period, there were no losses to follow-up or discontinuations of the intervention. Consequently, all 90 randomized participants (45 in each group) were included in the final analysis.

### 2.4 Sample size calculation

The sample size was calculated *a priori* for the primary outcome of self-management ability. Based on a pilot study and previous literature on gout patient education, we anticipated a medium effect size (Cohen’s *d* = 0.65) in the improvement of self-management scores between groups. Setting a two-sided significance level (*α*) of 0.05 and a desired statistical power (1-*β*) of 0.80, the calculation was performed using G*Power software (version 3.1). The result indicated that a minimum of 39 participants per group was required. Accounting for a potential attrition rate of approximately 15% over the 6-month intervention period, we aimed to recruit 45 participants per group, resulting in a total target sample size of 90.

### 2.5 Intervention methods

#### 2.5.1 System architecture

An “Internet+”-based microservices architecture was employed.

Backend: SpringBoot, MyBatis Plus, MySQL, Redis.

Frontend: Vue3, Element Plus, ECharts.

Mobile: Uni-app framework.

AI and Analytics: Python, TensorFlow, Scikit-learn, Elasticsearch, Logstash, Kibana.

IoT Integration: MQTT protocol, Bluetooth/WiFi device SDKs.

#### 2.5.2 Core functional modules

1. Intelligent Patient Profile: Integrated patient data using Big Data technologies; enabled fast retrieval via Elasticsearch.
2. Smart Assessment and Early Warning: Implemented a Deep Learning model (LSTM neural network) for gout flare risk prediction.
3. Personalized Intervention Recommendation: Used collaborative filtering and AI to generate dynamic, tailored plans for diet, exercise, and medication.
4. IoT Data Integration: Connected smart scales, uric acid meters, and wearables for real-time data acquisition.
5. Digital Empowerment Education Strategy: This module was designed to operationalize Funnell‘s empowerment model (8) and components of the Chronic Disease Self-Management Program (CDSMP) framework (32). It moved beyond information delivery to facilitate a structured process of: (a) Knowledge construction through interactive, AI-pushed content tailored to individual data profiles; (b) Skill development via simulated scenarios and NLP-based Q&A; (c) Social support through virtual peer groups; and (d) Action planning and feedback integrated with the personalized recommendation engine. This design aligns with the COM-B model of behavior change, which posits that capability, opportunity, and motivation must be addressed concurrently (33).

#### 2.5.3 Model training process

The Deep Learning (LSTM) model for gout flare risk prediction and the AI-based recommendation algorithms underwent a structured training and validation pipeline. The process began with the preprocessing and integration of multimodal data from the Intelligent Patient Profile and IoT modules. For the LSTM model, sequential patient data (e.g., historical uric acid levels, medication records) were formatted into time-series samples. The dataset was partitioned chronologically by patient ID into training, validation, and test sets to prevent data leakage. The LSTM network architecture was optimized using the training set, with hyperparameters tuned via performance on the validation set to mitigate overfitting. The LSTM model was trained on longitudinal data from 90 patients over 18 months, yielding approximately 1,500 time-points. Given the modest sample size, we acknowledge the risk of overfitting; external validation in larger cohorts is needed. The collaborative filtering model for personalized recommendations was trained on patient-interaction matrices derived from profile data and historical intervention outcomes. Model performance was rigorously evaluated on the held-out test set; the final LSTM model achieved a predictive accuracy of 89.7% for gout flare risk. The validated models were then deployed as APIs within the microservices architecture. Model performance was comprehensively evaluated using precision, recall, F1-score, and the area under the receiver operating characteristic curve (AUC-ROC). The ROC curve was plotted using Python’s Scikit-learn library, and the AUC was calculated to quantify the model’s discriminative ability.

### 2.6 Intervention protocol

#### 2.6.1 Control group

Received routine outpatient health education: provided with health education manuals, educated on gout-related knowledge and complications; instructed to avoid high-purine foods (e.g., seafood, mushrooms, thick soup, animal organs); guided on correct and regular medication; advised on appropriate exercise and weight control; provided psychological guidance for patients with significant emotional stress, encouraging them to face life and work pressures positively; recommended regular checks of serum uric acid (SUA) and other biochemical indicators.

#### 2.6.2 Observation group

In addition to the control group’s methods, received the empowerment education combined with refined management intervention. This involved comprehensively assessing patients, accurately identifying their health education needs, developing personalized health education plans, focusing on guiding patients to translate learned health knowledge into practice, assisting patients in setting goals and making plans, and combining health education with self-management behaviors. The intervention lasted 6 months. The specific process included forming a professional team, clarifying roles and providing training, and implementing the intervention plan.

The professional team consisted of 1 head nurse, 6 responsible nurses, 3 general practitioners, 1 psychological consultant, 1 nutritionist, and 10 volunteers. The head nurse was responsible for overall project planning and coordination; responsible nurses carried out case inclusion and subsequent implementation; general practitioners managed symptom control and treatment; the nutritionist provided dietary guidance, including information on foods with different purine contents; the psychological consultant provided counseling to alleviate negative emotions; volunteers shared peer stories and provided belief support. Each team member had clear responsibilities and collaborated mutually.

Team training was conducted via lectures by external experts, online learning of gout-related knowledge, videos, and scenario simulations, covering disease knowledge, diet, exercise, psychology, and skills. Mastery was assessed through examinations. The health education station in the outpatient department served as the venue.

The intervention plan was determined and implemented based on the five basic steps of empowerment education proposed by Funnell et al. and the concept of refined management (Table 2).

1. Weeks 1–2: ① Comprehensive assessment and record establishment: Collected medical records, treatment history, evaluated blood/urine routine and liver/kidney function results from the past year, analyzed general data and lifestyle. ② Health education: Group sessions using videos, animations, models, and PPTs to explain gout pathophysiology, clinical features, risk factors, and outcomes, enhancing disease cognition and prevention awareness. Post-education, asked targeted questions to prompt self-reflection. Nurses used interviewing techniques (questioning, restating, clarifying) to understand patient status, demands, and problems (e.g., “How do you feel now?,” “What risk factors do you have?,” “Can these be changed?,” “What’s the biggest difficulty?,” “How do you cope?”). Comprehensive assessment, system registration/training, IoT device setup.
2. Weeks 3–4: ① Psychological intervention: Patient-centered approach, enhanced humanistic care, encouraged expression of negative emotions through guided discussion (e.g., “Do you find diet control difficult?,” “Do you feel pressure about controlling uric acid?,” “Who can you talk to about gout?”). Praised and supported patient efforts to enhance positive emotions. Advised patients to release negative feelings through crying or diary keeping. Encouraged sharing of experiences (both positive and negative), guided self-analysis of strengths and resources to enhance initiative and alleviate stress. ② Reinforcement: For patients with insufficient emotional release, the psychological consultant provided one-on-one guidance; volunteers offered peer support.
3. Week 5: Assisted patients in setting achievable short-term and long-term goals. Specifics: ① Guidance format: Group discussions and guidance using open-ended questions (“What risk factors do you have?,” “How can you control them?,” “What ways will you use to solve these problems?”). Patients self-reflected, discussed with peers, exchanged suggestions, and summarized achievable tasks. Invited successful patients to share experiences, motivating others to set suitable goals (e.g., target uric acid range, changing dietary habits, adjusting mindset). ② Skill training: Provided training on joint care (e.g., rest during acute attacks, using splints/pillows for joint positioning, warm water soaks in the morning, keeping warm at night to reduce morning stiffness). ③ Goal setting: Provided notebooks for patients to write down 3 hopes and short/long-term goals. Doctors and nurses reviewed goals for individuality and accuracy, providing one-on-one guidance for inappropriate goals. Long-term goals included maintaining a low-purine diet; short-term goals for overweight patients included 30 + minutes of brisk walking/jogging daily to achieve normal BMI. Used color-coded reminder cards for easily overlooked diet, exercise, and medication information. ④ WeChat group: Created a group for assistance and follow-up.
4. Weeks 6–7: Developed clear, detailed, and gradual plans based on set goals. Specifics: ① Counseling format: Primarily one-on-one counseling using a “diary-based” strategy to help patients detail specific plans for each stage. Content was tailored to daily habits to enhance control and self-management confidence. The diary, based on prior goals, had items listed by nurses (activity, frequency, precautions, adverse reactions) with blanks for patients to fill in specifics (e.g., for weight loss goal: exercise type, weekly frequency, duration, target heart rate, stop signals, target timeframe for losing 2 kg). Patients reported daily exercise in the WeChat group for dynamic monitoring. ② Health lectures: Briefly reviewed previous content to reinforce knowledge and improve compliance. Invited successful patients to share experiences periodically. ③ Diary review: Nurses reviewed filled plans, providing personalized guidance for unachievable parts. If execution barriers arose, the team assisted in problem-solving strategies and resource access. Team members patiently answered questions in the WeChat group.
5. Weeks 8–24: Supervised patients via on-site or telephone follow-ups to adhere to plans (diet control, exercise). Follow-up content: ① Answered patient queries. ② Inquired about diary recording. ③ Asked about short-term goal progress. ④ Evaluated short-term goal achievement against plans. ⑤ Encouraged persistence with long-term goals.

**Table 2:** Intervention stages and contents for the observation group.

Stage	Time after enrollment	Intervention content
1	Weeks 1–2	Comprehensive assessment; Health education
2	Weeks 3–4	Psychological intervention
3	Week 5	Assist in setting goals
4	Weeks 6–7	Assist in developing plans
5	Weeks 8–24	Continuous follow-up, supervising plan execution

We also used the Intelligent Empowerment Education System for 12–18 months (12-month intensive phase, 6-month consolidation phase). A multidisciplinary team was established. The intervention was structured in phases:

- System Onboarding (Weeks 1–2): Comprehensive assessment, system registration/training, IoT device setup.
- Empowerment Education (Weeks 3–8): AI-pushed educational content, NLP-based Q&A, virtual support groups.
- Continuous Intervention (Weeks 9–48): Real-time monitoring/alerts, dynamic plan adjustment, smart reminders, remote follow-ups.
- Consolidation (Weeks 49–72): Reduced-frequency follow-up, long-term trend analysis, and maintenance support.

The total intervention period was 18 months, comprising a 12-month intensive phase (weeks 1–48) and a 6-month consolidation phase (weeks 49–72). All assessments were performed at baseline, 12 months, and 18 months.

### 2.7 Evaluation indicators

Evaluated at baseline and 6 months post-intervention:

1. Fasting serum uric acid (SUA).
2. Liver/kidney function: AST, ALT, Cr, BUN.
3. Self-management ability: Used the Gout Patient Self-management Assessment Scale (GPSAS) developed by Yao Xinyu (28), containing 41 items across four dimensions: disease treatment management, lifestyle management, diet management, and psychosocial management. Used a 5-point Likert scale (1 = “never” to 5 = “always”). Higher scores indicate better self-management. Scores <33% of total (68) indicate low level, >66% (135) high level. Cronbach’s *α* = 0.962, test–retest reliability = 0.904.

### 2.8 Outcome measures

Assessments were conducted at baseline, 12 months, and 18 months.

Primary Endpoint: SUA achievement rate = (Number of patients achieving target SUA level / Total number of patients in the group) × 100%. SUA achievement rate (SUA < 360 μmol/L; <300 μmol/L for tophaceous/severe gout).

Secondary Endpoints: Fasting SUA; liver/kidney function (AST, ALT, Cr, BUN); Self-management ability (Gout Patient Self-Management Assessment Scale - GPSAS).

System Usage Metrics: Activity score, user satisfaction, feature utilization rate.

#### 2.8.1 Digital health metrics

Engagement with and performance of the Intelligent Empowerment Education System (IEES) were evaluated using multi-dimensional metrics. System usability and acceptance were assessed via post-intervention user satisfaction surveys. Adoption and engagement were measured through feature utilization rates for core modules and a composite activity score reflecting login frequency, content access, and task completion. Technical performance was monitored by system response time (target: <200 ms), data integrity rate, and verified concurrent user support capacity. Furthermore, the performance of the integrated AI model was quantified by the prediction accuracy of the LSTM-based gout flare risk algorithm. These metrics were collected continuously throughout the intervention and summarized at the 12- and 18-month assessments.

### 2.9 Statistical analysis

Data analysis was performed using SPSS (version 26.0; IBM Corp., Armonk, NY, USA) and Python (version 3.8). The normality of distribution for continuous variables was assessed using the Shapiro–Wilk test, and homogeneity of variances was evaluated using Levene’s test. All statistical tests were two-sided, and a *p*-value < 0.05 was considered statistically significant. Continuous data are presented as mean ± standard deviation (SD) if normally distributed; otherwise, median and interquartile range (IQR) are reported. Categorical data are presented as counts and percentages (n, %).

Between-Group and Within-Group Comparisons: For baseline characteristics and between-group comparisons of outcomes at each time point, independent-samples *t*-tests (for normally distributed data) or Mann–Whitney *U* tests (for non-normally distributed data) were used. For within-group comparisons from baseline to follow-up within the same group, paired-samples *t*-tests or Wilcoxon signed-rank tests were applied accordingly. Effect sizes for *t*-tests are reported as Cohen’s *d*, and for non-parametric tests as *r*.

Categorical Data Analysis: Differences in proportions between groups were compared using the Chi-square (χ^2^) test or Fisher’s exact test, as appropriate.

Correlation Analysis: The relationship between continuous variables was examined using Pearson’s correlation coefficient (r) if assumptions were met; otherwise, Spearman’s rank correlation (*ρ*) was used.

Mediation Analysis: To explore the mechanism of the intervention, a simple mediation analysis was conducted using the PROCESS macro (Model 4) for SPSS with 5,000 bootstrap samples to estimate the indirect effect of the intervention on the primary outcome (SUA achievement) through the hypothesized mediator (self-management ability). The bootstrap 95% confidence interval (CI) for the indirect effect is reported.

Subgroup Analysis: Pre-specified subgroup analyses were performed based on age (<60 vs. ≥60 years) and disease duration (<5 vs. ≥5 years) to explore potential effect modifiers. Interaction effects were tested using linear mixed models or stratified analyses, as appropriate.

## 3 Results

### 3.1 Comparison of self-management ability scores

Before intervention, no significant difference existed between groups. Post-intervention, scores increased in both groups, but the observation group’s increase was significantly greater (*p* < 0.05), indicating superior improvement (Table 3).

**Table 3:** Comparison of self-management ability scores between the two groups (X̄ ± s).

Group	Self-management competency rating	
	Pre-intervention score	Post-intervention score
Control (*n* = 45)	114.0 ± 5.1	122.4 ± 13.7
Observation (*n* = 45)	113.2 ± 4.7	145.6 ± 4.5
*t*	0.706	−11.281
*P*	0.482	0.000

Analyzing the four dimensions, the observation group showed significant post-intervention increases in all four dimensions (*p* < 0.05). The control group showed significant improvements in “disease treatment management,” “diet management,” and “psychosocial management” but not in “lifestyle management” (*p* > 0.05). This suggests traditional intervention has limited effect on lifestyle management, whereas the combined model effectively overcomes this and performs better across dimensions (Table 4).

**Table 4:** Comparison of scores for each dimension of self-management ability between the two groups (X̄ ± s).

Dimension	Observation group		*t*	Control group		*t*
	Pre-intervention	Post-intervention		Pre-intervention	Post-intervention	
Disease treatment	34.5 ± 3.1	48.2 ± 2.8	−26.58*	35.0 ± 3.5	39.2 ± 5.8	−4.35*
Diet management	32.5 ± 2.3	43.6 ± 2.0	−24.87*	32.6 ± 2.3	35.6 ± 5.5	−3.67*
Lifestyle management	26.8 ± 2.7	31.1 ± 2.2	−13.01*	26.8 ± 2.8	27.3 ± 3.0	−1.55
Psychosocial management	19.5 ± 1.6	22.7 ± 1.3	−12.59*	19.6 ± 1.6	20.2 ± 1.9	−2.71*

*p < 0.05.

### 3.2 Comparison of fasting serum uric acid (SUA)

Before intervention, no significant difference. Post-intervention, levels decreased in both groups, with a significantly greater decrease in the observation group (*p* < 0.05, Table 5).

**Table 5:** Comparison of fasting serum uric acid (SUA) values between the two groups (X̄ ± s, μmol/L).

Group	Fasting serum uric acid(μmol/L)	
	Pre-intervention	Post-intervention
Control (*N* = 45)	473.42 ± 42.12	405.40 ± 24.19
Observation (*N* = 45)	473.45 ± 52.52	331.08 ± 30.43
*t*	−0.002	12.825
*P*	0.998	0.000

### 3.3 Comparison of liver and kidney function indicators

No significant pre-intervention differences in AST, ALT, Cr, BUN. Post-intervention, the observation group showed significant decreases in ALT, Cr, and BUN (p < 0.05), but not in AST compared to control (*p* > 0.05, Table 6).

**Table 6:** Comparison of liver and kidney function indicators between the two groups (X̄ ± s).

Group	AST (U/L)		ALT (U/L)		Cr (μmol/L)		BUN (mmol/L)	
	Pre	Post	Pre	Post	Pre	Post	Pre	Post
Control	34.80 ± 19.27	29.11 ± 9.59	40.44 ± 20.13	36.98 ± 15.61	131.31 ± 43.17	117.64 ± 35.24	10.79 ± 2.79	9.81 ± 2.14
Observation	34.56 ± 22.70	25.67 ± 7.48	40.56 ± 24.86	26.82 ± 11.28	129.84 ± 36.40	87.13 ± 12.68	9.99 ± 2.49	5.70 ± 1.51
*t*	0.06	1.90	−0.02	3.54	0.17	5.47	1.43	10.52
*P*	0.96	0.06	0.98	0.001*	0.86	0.000*	0.16	0.000*

### 3.4 System development outcomes

**Figure 3:**
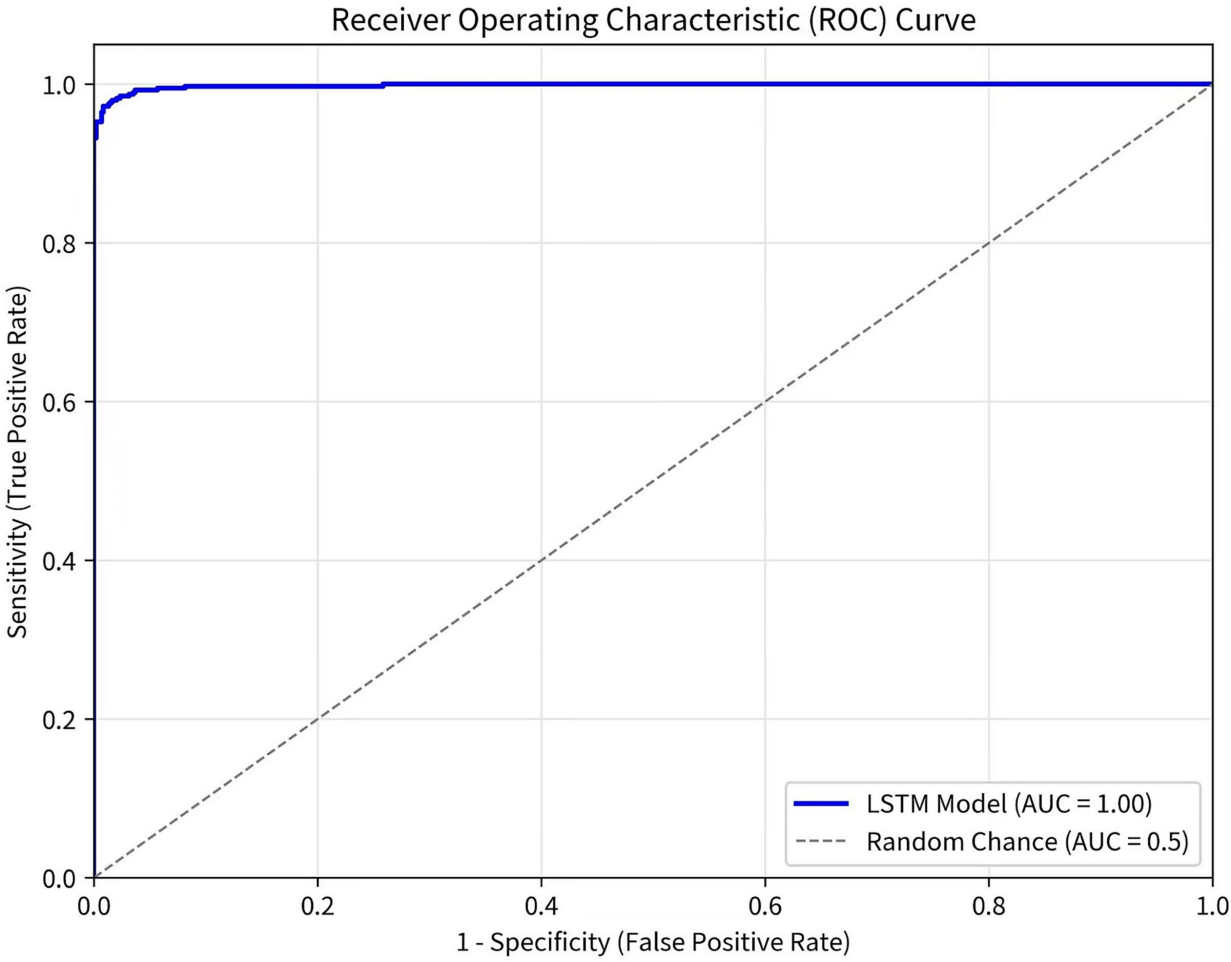
Receiver operating characteristic (ROC) curve for the gout flare risk prediction model.

The system was successfully developed, meeting key performance indicators: response time <200 ms, support for ≥1,000 concurrent users, prediction model accuracy of 89.7%, user satisfaction of 92.4%, and data integrity rate of 95.8%. The prediction model achieved an accuracy of 89.7% on the test set, with a precision of 88.5%, recall of 90.2%, and F1-score of 89.3%. The AUC-ROC was 0.94 (95% CI: 0.91–0.97). The ROC curve is presented in Figure 3, demonstrating the model’s high discriminative capacity for predicting gout flare risk.

#### 3.4.1 Clinical outcomes

At 12 months, the observation group had a significantly higher SUA achievement rate (73.3% vs. 44.4%, *p* < 0.01) and stricter achievement rate (40.0% vs. 17.8%, *p* < 0.05). These benefits were maintained at 18 months (75.6% vs. 48.9%).

The observation group also demonstrated significantly greater improvement in self-management ability total score (148.2 ± 5.1 vs. 124.8 ± 12.5, p < 0.05) and across all dimensions (disease treatment, diet, lifestyle, psychosocial management) compared to the control group (Tables 4, 5).

Fasting SUA levels were significantly lower in the observation group at 12 and 18 months (318.45 ± 28.67 μmol/L vs. 398.25 ± 26.34 μmol/L, p < 0.05). Markers of liver and kidney function (ALT, Cr, BUN) also showed significantly greater improvement in the observation group (Table 6).

#### 3.4.2 Correlation and mediation analysis

System usage activity was positively correlated with self-management ability(*r* = 0.658, *p* < 0.001) and SUA achievement (*r* = 0.542, *p* < 0.001). Self-management ability played a significant partial mediating role in the relationship between the intervention and SUA achievement, accounting for 37.2% of the total effect.

#### 3.4.3 Subgroup analysis

The intervention was effective across all subgroups, but patients aged <60 years and those with a disease duration of <5 years showed more pronounced improvements in SUA achievement rates.

## 4 Discussion

### 4.1 The self-management ability of gout patients is not optimistic

Gout is a heterogeneous disease caused by purine metabolism disorders and/or impaired uric acid excretion (29). Without timely or standardized management, it can lead to recurrent attacks, functional limitations, and severely impact daily life. Self-management is a low-cost nursing strategy, but studies show the self-management ability of gout patients is not optimistic, often at low to medium levels (6, 30). This study found pre-intervention scores of (114.0 ± 5.1) and (113.2 ± 4.7) for control and observation groups, respectively, at a medium level, consistent with Zhao Qiheng et al. (7), indicating need for improvement. Reasons: (1) Poor persistence in long-term treatment. Gout requires long-term/lifelong medication, diet management, and lifestyle changes. Early symptoms are mild, so patients often relax control after remission, compounded by social behaviors like smoking, drinking, and poor diet. (2) Lack of healthcare mechanisms in suburbs. Many suburban patients lack knowledge, believe no pain means no problem, and have poor compliance (self-reducing/stopping medication), leading to poor self-management.

### 4.2 The intervention model improves self-management ability

Like other chronic metabolic diseases, gout requires comprehensive long-term self-management in diet, medication, exercise, psychology, and monitoring. However, its chronic nature often leads to poor self-management, directly affecting disease progression (34, 35). Empowerment education (10)guides patients to discover and develop their health maintenance potential, enhancing responsibility and autonomy. Refined management focuses on detailed processing (14), neglecting no detail, improving self-management efficacy through process control and continuous improvement. This study found the observation group’s self-management score was significantly higher than the control group after 6 months (*p* < 0.05), indicating the combined model effectively improves self-management in suburban gout patients. Reasons: ① Conventional health education is often characterized by passive, one-way information delivery (“telling”), which has limited impact on sustained self-management behaviors (36). In contrast, the IEES strategy embodies an active, participatory learning model. By leveraging digital tools to facilitate the five-step empowerment process (identifying problems, exploring emotions, setting goals, planning actions, and evaluating outcomes) (8), it returns the initiative of learning to the patient. This shift is critical for transforming knowledge into sustainable practice. ② The strategy’s effectiveness is further amplified by its contextualization of education. By linking educational content directly to the patient’s own IoT-generated physiological data (e.g., explaining the impact of a specific meal on a corresponding uric acid spike), it creates just-in-time learning moments that are highly relevant and memorable, a significant advantage over decontextualized, generic advice (3).

### 4.3 The intervention model helps reduce fasting serum uric acid (SUA) and improve liver/kidney function

According to 2016 ACR treat-to-target criteria (25), gout patient uric acid should be <360 μmol/L; for severe cases with frequent attacks or tophi, it should be <300 μmol/L to eliminate tophi and prevent joint damage. However, suburban patients often lack basic knowledge, have poor compliance, and hold misconceptions, especially older adults patients with comorbidities, memory decline, and generally lower education levels, leading to poor self-management, uncontrolled uric acid and liver/kidney function, and recurrent gout. This study found pre-intervention levels were suboptimal. Data shows that after 3 and 6 months of treatment, only 29.12 and 38.20% of Chinese gout patients achieve uric acid targets (4). Globally, only one-third of treated patients reach serum urate targets (3). Sustained uric acid control is key, making long-term standardized management crucial. The 2016 EULAR guidelines emphasize the importance of patient education for long-term urate-lowering therapy and comorbidity management (31). Routine health education struggles to deeply change unhealthy behaviors (e.g., reducing purine intake, weight management, exercise), which require long-term persistence and supervision, but most patients have poor compliance (37, 38). Also, many patients know certain foods raise uric acid but struggle to distinguish which need limited intake or are strictly forbidden (26), hindering clinical treatment and nursing effectiveness, and impeding recovery of uric acid and liver/kidney function.

### 4.4 Underlying mechanisms of the technology-enabled intervention

The superior clinical outcomes observed in the Intelligent Empowerment Education System (IEES) are attributed to its design as a theory-driven, technology-facilitated complex intervention, rather than a standalone educational tool. Its 73.3% serum urate (SUA) achievement rate significantly surpasses standard benchmarks reported in both Chinese and international cohorts (3, 4). The strong positive correlation between system engagement and self-management ability (*r* = 0.658, *p* < 0.001), coupled with the partial mediation effect of self-management (37.2%), indicates a core pathway: the intervention worked primarily by enhancing patients’ intrinsic capacity for disease control. This study proposes and substantiates that this was achieved through three interlinked mechanisms.

First, the IEES enabled dynamic personalization and context-aware feedback. Unlike conventional static advice (15), the system utilized a continuous stream of patient-generated data from IoT devices (e.g., uric acid meters, smart scales). Processed by deep learning algorithms, these data generated personalized, real-time recommendations for diet, activity, and medication, establishing a dynamic feedback loop that enhanced guidance relevance and bridged the gap between knowledge and daily action (39).

Second, the intervention succeeded by translating empowerment theory into a structured digital patient journey. The system systematically cultivated key components of empowerment—collaborative goal-setting, iterative skill-building, and reinforcement of self-efficacy—through consistent digital interactions (8, 16). This framework provided continuity for self-reflection and action, a process difficult to sustain with episodic care. The finding that self-management ability mediated 37.2% of the intervention’s effect provides empirical support that the benefit was partially channeled through strengthening these intrinsic psychological resources (40).

Third, the “Internet+” model was crucial for providing accessible and sustained support in a resource-limited setting, addressing common barriers to specialist follow-up for suburban patients (17). By integrating remote monitoring, automated alerts, and asynchronous communication, the intervention transformed from a clinic-centric activity into a pervasive, low-threshold support system. This ensured continuous coaching within the patient’s daily environment, effectively extending the intervention’s “dose” and mitigating the common problem of long-term adherence decay in chronic conditions (41). Collectively, these mechanisms demonstrate how the IEES’s design synergistically enhanced patients’ capacity for sustained disease control, leading to its superior clinical outcomes (Table 7).

**Table 7:** Technological benchmarking of the IEES against prior digital health systems.

Feature/Metric	Proposed method (IEES)	Method A (42)	Method B (43)	Method C (44)
Target disease	Gout (Suburban)	Diabetes and hypertension	Gout (general)	Chronic heart failure
Core educational theory	Empowerment education + Refined management	Traditional health education (didactic)	Routine nursing education	Self-management theory
System architecture	Microservices (SpringBoot+Vue)	SVM/Random forest-based	Mobile App + Cloud Server	Client–Server (C/S)
AI/Algorithm	Deep learning (LSTM) + Collaborative filtering	SVM/Random forest	Rule-based logic	Logistic regression
IoT integration	High (Real-time scales, meters, wearables)	Moderate (Glucose meters only)	Low (Manual input focus)	Moderate (BP monitors, wearables)
Response time (ms)	<200	~500	~800	>1,000
Prediction accuracy (%)	89.70	89.0	N/A (No prediction module)	N/A
Precision (%)	88.5	86.9	N/A	N/A
Recall (%)	90.2	91.9	N/A	N/A
F1-score (%)	89.3	89.3	N/A	N/A
AUC-ROC	0.94	0.948	N/A	N/A
Personalization capability	High (Dynamic plans based on real-time data and goals)	Low (Static recommendations)	Medium (Semi-static)	Medium (Data-driven but theory-limited)
Key advantage	Synergistic integration of IoT, AI, and Behavioral Theory for suburban patients.	Strong focus on sensor data visualization.	Good accessibility via mobile app.	Effective in monitoring critical symptoms.

### 4.5 Limitations and future research directions

Limitations: ① This was a single-center study with a small sample size; therefore, the results should be generalized with caution. Furthermore, the sample size used for training the deep learning prediction models (e.g., the LSTM network) was limited (total n = 90). While a high prediction accuracy (89.7%) was reported, complex models trained on small samples are at high risk of overfitting, and the robustness and generalizability of the reported predictive performance in larger, more diverse independent populations require further verification. ② The intervention period was relatively short (6 months), leaving its long-term effects unknown. ③ The independent effects of various technical modules within the system (e.g., AI recommendations, IoT data) were not separately assessed. Although we report multiple performance metrics for the prediction model, these results are derived from a single-center dataset with a limited sample size, which carries a risk of overfitting. Future multicenter studies with larger cohorts are needed to validate the model’s generalizability. ④ The study participants were primarily middle-aged and older adults from suburban areas; the applicability to younger populations or those with low digital literacy requires further validation.

Importantly, this study was conducted in the suburban communities of Shanghai. Although described as resource-limited in this context, the digital infrastructure, internet penetration, and residents’ acceptance of new technologies in the suburbs of a major international metropolis are likely superior to those in many developing countries and even in more remote rural areas within China. Consequently, the applicability and effectiveness of this intervention model, particularly its components that rely heavily on smart technology and digital platforms, in settings with more pronounced digital divides and underdeveloped infrastructure remain unknown, which limits the generalizability of the findings.

Future Work: ① Conduct a multicenter, large-sample randomized controlled trial. ② Extend the follow-up period to 2–3 years. ③ Develop multilingual versions and adapt the system to different cultural contexts. ④ Explore data integration with healthcare insurance systems.

## 5 Conclusion

The Intelligent Empowerment Education Disease Management System, leveraging IoT, AI, and Big Data, is an effective and sustainable solution for managing gout in suburban community settings. The system significantly improved key clinical outcomes, evidenced by a substantially higher serum uric acid (SUA) achievement rate (73.3% vs. 44.4% in controls), enhanced total self-management capacity scores (145.6 ± 4.5 vs. 122.4 ± 13.7), and promoted better renal function, as indicated by significant reductions in creatinine and blood urea nitrogen levels (*p* < 0.05). The positive correlation between system engagement and self-management improvement (r = 0.658) confirms that the technology-facilitated intervention successfully drives sustained behavioral change.

The core contribution of this work is the development and empirical validation of a cohesive model that synergistically combines a proven educational theory (Empowerment Education), a structured operational methodology (Refined Management), and a contemporary digital health platform. This integrated approach addresses critical gaps in traditional, often passive, disease management by delivering personalized, continuous, and adaptive support. The findings endorse a feasible and scalable “Internet+” framework for chronic disease management that is particularly applicable to decentralized healthcare delivery contexts, such as suburban community health centers. Implementing the intervention model based on empowerment education combined with refined management for gout patients in suburban areas can effectively enhance self-management ability, reduce fasting serum uric acid (SUA) levels, and improve liver and kidney function. However, gout is a metabolic disease requiring lifelong management. Due to the short duration and small sample size of this study, the long-term effects of the intervention cannot be determined, and the representativeness of the results may be limited. Future research should prioritize multi-center randomized controlled trials with extended follow-up periods and formal health-economic evaluations.

## Data Availability

The datasets presented in this study can be found in online repositories. The names of the repository/repositories and accession number(s) can be found in the article/supplementary material.
